# Supplementary material for: Source-to-sink coupling and spatiotemporal evolution during multiphase rifting: The Jurassic-Cretaceous Lishu fault depression, Southeastern Songliao Basin
Source: PLoS One. 2026 May 4;21(5):e0348267. doi: 10.1371/journal.pone.0348267 (PMC13138628; doi:10.1371/journal.pone.0348267)
Supplement: Attachment — Submitted filename: Response to Reviewers.docx [file pone.0348267.s003.docx]

**Response to Reviewers**

We sincerely appreciate the reviewers for their thorough evaluation of our manuscript and for providing insightful comments that greatly assisted us in improving the quality of the paper. We have carefully addressed each comment, and our detailed responses are provided directly below each corresponding remark in red text.Once again, we would like to express our gratitude for your time and constructive review of our work.

**Reviewer #1:**

1. The Introduction provides a rich contextual background; however, the paper may benefit from a clearer and more direct articulation of its scientific questions or hypotheses. Enhancing this aspect could help readers better appreciate the novelty and scope of the investigation.

Thank you very much for your valuable comments on my manuscript. Regarding your suggestions for improving the Introduction section, I fully agree that a clearer articulation of the scientific questions and hypotheses would help readers better understand the novelty and scope of this study. In the revised version, I have reorganized the Introduction to explicitly define the core scientific issues addressed in this work and to highlight the uniqueness of the research hypotheses. Specifically:

Clear statement of scientific questions:

At the beginning of the Introduction, I now provide a more explicit description of the specific scientific questions targeted in this study, as well as the existing gaps and debates in the current literature that motivate these questions.

Strengthening of research hypotheses:

Based on the stated questions, I have further elaborated on the rationale behind the proposed hypotheses and emphasized how they differ from previous studies. This revision clarifies both the theoretical significance and the practical value of the hypotheses.

Through these modifications, I aim to enhance readers’ understanding of the core content and innovations of the paper.

The details can be found in lines 89–100.

Once again, thank you for your insightful comments, and I look forward to any further feedback you may have.

2. The conclusions might benefit from a more concise structure with emphasis on the core findings, scientific contributions and broader implications. Please make further modification.

Thank you very much for your careful review of our manuscript and for the valuable comments you provided. We fully agree with your suggestion regarding the structure of the conclusion section and have made the corresponding revisions.

Specifically, we have streamlined the conclusion to better highlight the core findings, scientific contributions, and the broader implications of our study. By reorganizing the content, our aim was to present the central ideas of the manuscript in a clearer, more concise, and logically coherent manner.

In particular, the revised conclusion now emphasizes the following key points:

The major findings and innovations of the study;

The contributions of these findings to existing theories and methodologies;

The potential implications of the results for broader geological research and practical applications.

The relevant revisions can be found in lines 690–709.

We believe that these revisions have made the conclusion more focused and persuasive. Thank you again for your insightful suggestion, and we look forward to any further feedback you may have.

3. Heavy mineral assemblages and sandstone components are used to indicate provenance direction, where heavy minerals primarily reflect provenance sources, and clastic components of sandstone mainly indicate transport distance. During the description of provenance system evolution in each rifting phase, the interpretation of provenance evolution in different regions reflected by sandstone clastic components is insufficient. Further clarify the significance of sandstone components for provenance area analysis.

Thank you very much for your review and valuable comments on our manuscript. Regarding your concern that “the interpretation of provenance evolution in different regions based on sandstone clastic compositions is insufficient,” we have carefully re-examined the relevant sections and made substantial revisions and additions. Specifically:

Significance of sandstone clastic compositions for provenance analysis:

We have further clarified in the revised manuscript the importance of sandstone clastic components in provenance interpretation. The detrital composition of sandstone not only reflects the primary source areas but also provides insights into transport distance, degree of weathering, and depositional conditions. During the provenance evolution associated with different stages of rifting, variations in sandstone clastic components help identify spatial shifts in source areas. This is particularly relevant in a multiphase rift setting, where changes in provenance are closely linked to the tectonic evolution of the rift basin.

Interpretation of provenance evolution in different regions:

In the revised version, we have added more detailed explanations distinguishing the provenance evolution across different rift stages. By integrating variations in sandstone clastic components with the characteristics of heavy mineral assemblages, we discuss the dynamic changes in source areas, outline the provenance evolution patterns for each rifting stage, and explore their relationships with geological controls such as tectonic activity and climatic influences.

Expanded discussion:

To further strengthen the link between sandstone composition and provenance evolution, we have supplemented the manuscript with several key case studies illustrating how sandstone clastic compositions can indicate the evolution of different source regions. In addition, we have added corresponding figures and diagrams to better visualize the provenance changes across different developmental stages.

We hope these revisions adequately address your concerns and improve the manuscript. Thank you once again for your constructive suggestions, and we look forward to your further evaluation.

4. The study meticulously characterizes the "source system," "transport system," and "sedimentary system," but the correlation among the three subsystems is weak, and the coupling of provenance system-transport pathway-sedimentary system is not emphasized. In the discussion section, further clarify the coupling relationship and reflect it in the classified source-sink system types.

Thank you very much for your careful review of our manuscript and for the valuable feedback you provided. In response to the reviewer’s comments regarding the coupling relationships among the “source system,” “transport system,” and “depositional system,” we have thoroughly revised and supplemented the manuscript.

The specific revisions are as follows:

Enhanced discussion of source–transport–deposition coupling:

In the revised manuscript, we have strengthened the discussion in the Discussion section by explicitly clarifying the interconnections and coupling mechanisms among the source, transport, and depositional systems. In particular, we highlight the relationships among material supply from the source area, sediment-routing pathways, and depositional environments, as well as how these couplings vary under different geological conditions.

Revised classification framework and correlation analysis:

We have modified the original classification scheme and improved the correlation analysis among the subsystems to provide a clearer and more systematic framework.

These revisions aim to further clarify the linkages among the different subsystems and enhance the logical coherence and academic value of the manuscript.

Once again, we sincerely appreciate your constructive comments and suggestions. We look forward to any further feedback you may have.

5. It is recommended to remove the wells not mentioned in the text from Fig 1, and the reference for the stratigraphic column in Fig 2 must be cited from previous studies.

We sincerely appreciate the reviewer’s valuable comments on our work. In accordance with the suggestions provided, we have made the following revisions:

Regarding the wells shown in Fig 1:

As the reviewer correctly noted, several wells depicted in Figure 1 were not referenced in the manuscript. To ensure consistency between the figure and the text, we have removed these wells from Fig 1.

Regarding the reference sources for the stratigraphic columns in Fig2:

Following the reviewer’s suggestion, we have added and cited previous studies as the reference sources for the stratigraphic columns in Fig 2. This modification enhances the reliability and academic rigor of the figure.

We believe that these revisions have improved the overall quality of the manuscript. We sincerely thank the reviewer again for the constructive and insightful comments.

6. The Fig.17 might be refined for clarity and representation of the sedimentary models.

We sincerely appreciate the reviewer’s valuable comments. In response to the suggestions regarding the improvement of Figure 17, we have further optimized the figure to enhance its clarity and the effectiveness of sedimentary model representation. The specific revisions are as follows:

Enhanced image clarity:

We have adjusted the contrast and resolution of the figure to make key details more prominent, thereby facilitating clearer identification of the different sedimentary units.

Redesigned sedimentary model:

The layout of the sedimentary model has been redesigned to more intuitively illustrate the evolutionary process and hierarchical relationships within the model. The arrangement of legends and annotations has also been optimized to simplify the information displayed and reduce unnecessary redundancy, thereby improving overall readability.

We believe these revisions will help readers better understand the sedimentary model and further enhance the academic quality of the figure. Once again, we sincerely thank the reviewer for the careful evaluation and constructive suggestions provided.

7. Please keep the sedimentary terminology in consistent throughout the manuscript, which would improve clarity of the paper.

We sincerely appreciate the reviewer’s valuable comments. Regarding the issue of consistency in sedimentological terminology, we have carefully re-examined the entire manuscript and corrected all inconsistencies. Standardized and unified sedimentary terms are now used throughout the text to ensure greater clarity and professional accuracy. These revisions have been implemented in the revised manuscript, and we thank the reviewer for drawing our attention to this important point.

Reviewer #2:

1. Whether it is the abstract, introduction, or discussion section, the manuscript is confined to a single rift basin in the southeastern part of the Songliao Basin. It discusses the deficiencies in the research on S2S, and its global significance needs to be enhanced. Furthermore, there are numerous research results on S2S in continental basins, covering various research fields. The manuscript lacks sufficient scientific issues and innovative points.

Thank you very much for your thorough review and valuable comments on my manuscript. Following the reviewer’s suggestions, I have revised the manuscript as follows:

(1) Enhancing the global significance of the study:

To broaden the impact of this research, I have explicitly articulated the global scientific relevance of studying the southeastern rift basins of the Songliao Basin in the Abstract, Introduction, and Discussion sections. By incorporating an S2S (source-to-sink)–based case analysis of the Songliao Basin, I have compared the findings with other continental basins and emphasized the universal relationships among fault activity, tectonic evolution, and sedimentary processes in rift basins worldwide. Specifically, I highlight how the S2S system of the Songliao rift provides meaningful insights applicable to sediment routing and source–rock distribution in other basins with similar tectonic settings, especially regarding sediment dispersal and evolutionary patterns under different tectonic regimes.

(2) Strengthening the emphasis on scientific innovations:

I have revised the manuscript to better highlight the innovative application of S2S concepts within the context of source-to-sink system analysis. This includes detailed characterization of fault activity and sediment routing relationships, which reveal how newly developed fault networks within the Songliao rift system influence sediment distribution and reservoir development. I propose a temporal–spatial evolution mechanism of S2S processes in rift basins and discuss the impact of fault activity at different stages on sediment supply—an issue that has not been sufficiently addressed in previous literature. To address gaps in existing S2S research, particularly the coupling between multistage rifting and sedimentary processes, I introduce a new integrative research framework that combines seismic interpretation, core observation, sequence stratigraphy, and structural analysis, providing new perspectives and methodologies for understanding S2S evolution. Additionally, I broaden the geological implications by comparing the Songliao Basin with the East African Rift, offering new insights into the tectono-sedimentary evolution of rift basins on a global scale.

(3) Reinforcing the discussion of the source-to-sink system:

I have expanded the theoretical background and practical implications of S2S concepts, clarifying how the timing and magnitude of fault activity influence sediment generation, routing, and deposition within rift basins. By incorporating recent research findings, I further demonstrate how the S2S framework developed for the Songliao Basin can contribute to advancing studies of sedimentary systems and tectonosedimentary evolution in similar rift settings.

2. In the introduction, the manuscript devotes considerable space to elaborating on the significance of S2S research, as well as the differences in the configuration of S2S between marine basins and continental basins. However, the discussion on the existing issues in the current global research S2S in continental rift basins, as well as the scientific significance of conducting source-to-sink system research using the Songliao Basin as an example, is clearly insufficient.

Thank you very much for your valuable suggestions regarding the Introduction section. Following your advice, I have revised the relevant content and have specifically strengthened the discussion of the major issues in global S2S (source-to-sink) research within continental rift basins, as well as the scientific significance of using the Songliao Basin as a representative example.

In the revised Introduction, I have provided a more detailed discussion of the key challenges currently faced in S2S studies of continental rift basins worldwide, particularly those related to data acquisition and interpretation. The complex tectonic settings, sedimentary evolution, and their interactions with syn-depositional fault activity often result in pronounced spatial variability within rift-basin S2S systems, thereby introducing significant heterogeneity to research outcomes. In addition, regional geological variations, uneven lithofacies distributions, and the lack of integrated datasets contribute to the complexity of S2S models in continental rift basins, and a unified conceptual or theoretical framework has yet to be established.

The Songliao Basin, as a typical continental rift basin in China with a unique geological background and a well-preserved record of multi-phase rifting, provides an ideal natural laboratory for S2S investigations. Conducting S2S research in the Songliao Basin offers valuable regional constraints for understanding the characteristics and evolution of source-to-sink systems in rift settings globally and facilitates a deeper examination of fault–sediment system interactions under multi-stage extensional regimes. Such studies not only fill the existing research gap in S2S analysis within this basin but also contribute meaningful insights for hydrocarbon exploration and tectono-sedimentary evolution in other rift basins.

Through integrated geological, geophysical, and sedimentological analyses, this study further elucidates the spatiotemporal distribution patterns of S2S systems in the Songliao Basin, with particular emphasis on the coupling relationships between fault activity and sedimentary systems during different rifting stages. Compared with previous works, the present study approaches the problem from an S2S-interaction perspective and explores the dynamic mechanisms that govern source-to-sink evolution, thereby providing a new conceptual angle for understanding mass transfer processes in rift basins.

The details can be found in lines 89–100.

I have revised the Introduction accordingly and added the relevant background information and discussion. Thank you again for your constructive comments, and I look forward to your further guidance.

3. The discussion section needs improvement in the following aspects: (1) It should conduct in-depth comparative analysis based on research at the forefront of the discipline and in conjunction with previous studies. (2) There is an excessive focus on process description, with a lack of exploration into mechanisms. For instance, the manuscript conducts quantitative analysis, but what are the dynamic implications of these analysis results for the differential development of S2S in rift basins across different periods? (3) There is a lack of necessary discussion on the broader implications of the research findings, particularly in terms of global significance.

Thank you very much for your valuable comments. Following your suggestions, we have revised the Discussion section as follows:

(1) Comparative analysis: We have incorporated an in‐depth discussion of recent advances in related research fields and conducted a comparative analysis between our study and source-to-sink (S2S) evolutionary patterns documented in other rift basins. This revision highlights the key differences in S2S evolution under varying structural settings.

(2) Mechanistic insights: To further address the mechanism-oriented aspects of your comments, we have elaborated on the dynamic processes controlling differential S2S development in rift basins, with particular emphasis on the profound influence of tectonic activity and depositional environments on source-to-sink evolution.

(3) Global significance: We have expanded the discussion on the broader geological implications of S2S evolution, especially regarding the general applicability of rift-basin S2S models to global geological studies and resource exploration.

4. The repetitive expressions in 5.2 and 5.3 under discussion can be merged. In addition, it may be more appropriate to place the quantitative analysis of the S2S in the results section.

We sincerely appreciate the reviewer’s thorough evaluation of our manuscript and the valuable comments provided. In response to the concerns raised, we have made the following revisions:

Consolidation of repetitive content in Sections 5.2 and 5.3:

We have carefully re-examined the content of these two sections and merged the overlapping descriptions to improve conciseness and logical coherence. After consolidation, the remaining text was further refined to ensure that the discussion is clearer and more streamlined.

Placement of the quantitative S2S analysis in the Results section:

We fully understand the reviewer’s suggestion. However, this part of the analysis involves not only data presentation but also a broader discussion of its general implications. Keeping it within the Discussion section better highlights its interpretative significance and maintains a more coherent overall structure of the manuscript. Therefore, we believe it is more appropriate to retain this content in the Discussion.

We sincerely thank the reviewer again for the constructive feedback. We are confident that these revisions have enhanced the overall quality of the manuscript. Should further modifications be required, we will be pleased to address them.

5. When calculating the fault activity rate, it is necessary to clarify the basis and source for obtaining the deposition age of the strata.

We sincerely appreciate the reviewer’s valuable comments. Regarding the concern that “the basis and sources of the stratigraphic ages used to calculate fault activity rates should be explicitly clarified,” we have now provided additional explanation in the revised manuscript.

Specifically, the stratigraphic ages employed in this study are primarily derived from previously published literature. We incorporated regional and adjacent stratigraphic age datasets to ensure consistency and reliability of the chronological framework. These basis and data sources have been described in detail in Section 3.2.2 of the revised manuscript, where we also cite the relevant references to clearly demonstrate the provenance of the sedimentary age information used in our analysis.

We again thank the reviewer for the careful and constructive review and look forward to any further comments.

6. Regarding the analysis of tectonic paleogeomorphology, the labeling of major faults is not clear. Many gullies are depicted (mostly with semi-transparent labeling) in Figure 6, but their morphology is not clear from the plan view, and many areas with clearly developed gullies (such as the northeastern part of the figure) are not depicted.

Thank you very much for your thorough review and valuable comments. Regarding your suggestions on the analysis of the structural paleogeomorphology and the fault annotations in Figure 6, we have further revised and optimized the figure as follows:

Clarification of major fault annotations:

We have re-labeled the key faults in Figure 6 to make their positions and geometries more clearly identifiable.

Improvement of gully morphology clarity:

The depiction of gullies has been refined by enhancing the display of local details, adjusting the transparency of semi-transparent labels, and adding more precise annotations. These adjustments improve the clarity of the map layers and labels, ensuring that the morphology of the gullies is more easily recognized in plan view.

Explanation for the previously unillustrated area:

As per your suggestion, we re-examined the unannotated gully region in the northeastern part of Figure 6. The apparent gullies were artifacts generated during the paleogeomorphic restoration process due to software limitations. For each gully-like feature, we have reviewed the corresponding seismic profiles. Although some gullies appear in the plan-view restoration, they do not correspond to actual topographic relief in the seismic data.

Once again, we sincerely appreciate your careful feedback on our work.

7. Generally, we conduct S2S division by comprehensively considering the configuration relationship between the catchment (water system), transport system, and depositional system. However, the manuscript lacks basis and principles in the classification of S2S types. From the textual description of the manuscript, the type of transport system may serve as an important basis for classification, which raises some debatable issues: some of the source-to-sink system types (such as Convergent) classified in the manuscript may include other types; similarly, if the geomorphic characteristics of the provenance are not considered, such as the "Along-strike Slope type" classified in the gentle slope zone, it is impossible to explain the differences in S2S under multiple provenance-valley-deposition configurations, thereby affecting the prediction of sediment dispersion and favorable reservoir sand bodies.

Thank you very much for your valuable comments on our manuscript. Regarding the issue you raised about the classification of S2S types, we have carefully considered your suggestions and made corresponding revisions.

On the insufficient basis and criteria for S2S classification:

We acknowledge that in the original version, the rationale and principles underlying the classification of S2S types were not adequately articulated. In response, we have removed this section from the revised manuscript. We believe that even without explicitly classifying S2S types, the evolution of the source-to-sink system in the multistage rift basin of the Lishu Fault Depression can still be discussed in sufficient detail.

We hope that these revisions address the issues you identified and improve the scientific rigor and logical clarity of the manuscript. Thank you again for your constructive comments, and we look forward to your further review.

8. In the analysis of source-to-sink systems, the transport systems related to faults generally include transition zones and fault troughs. Among them, fault troughs are channels or depressions controlled by faults, including single-fault troughs, co-trending double-fault troughs, and reverse double-fault troughs. I don't quite understand the true intention of the manuscript to use fault combinations as transport systems. Moreover, in the analysis of fault combinations, there is a lack of interpretation of fracture combinations in different depositional periods, as shown in Figures 10b and c. Using schematic diagrams to express seems inappropriate.

Thank you very much for your valuable comments on our manuscript. In response to your concerns regarding the use of fault assemblages in the source-to-sink (S2S) analysis, we have carefully reconsidered the relevant sections and made corresponding revisions and clarifications in the manuscript.

First, concerning your point about the “true intention of using fault assemblages as transport systems,” our rationale for employing fault assemblages was to better characterize the diverse interactions and transport behaviors of faults under complex structural conditions. We fully acknowledge that, traditionally, fault troughs—including single-fault troughs, synthetic dual-fault troughs, and antithetic dual-fault troughs—are widely used to describe transport pathways controlled by one or multiple faults. However, in our study area, fault troughs have been categorized as a specific type of valley landform, and basin-margin fault troughs are interpreted as the structural extension of valleys. For this reason, fault troughs are not further elaborated in this study. Additionally, we agree that the term “fault assemblage” may introduce ambiguity. Therefore, following your suggestion, we have replaced “fault assemblage” with “fault-step zone (fault–step belt),” which more accurately defines the sediment-routing characteristics of this transport pathway.

Regarding your comment that the use of a schematic diagram may be inappropriate, we have revised the corresponding figure accordingly and removed the schematic illustration as suggested.

We greatly appreciate your insightful comments and have revised the manuscript in accordance with your recommendations. Thank you again for your valuable time and constructive feedback.

9. The expression of structural (fault) transfer zones is relatively vague, and the supporting maps cannot illustrate the characteristics of the transfer zones. Based on the interpretation of faults and the reconstruction of paleogeomorphology in the study area, the manuscript needs to first clarify which areas in the study area have transfer zones, then confirm the types of transfer zones, and finally, for the main types of transfer zones, demonstrate the relationship between faults from both plan and profile perspectives.

We sincerely appreciate your valuable comments on our manuscript. In response to your concerns regarding the “relatively ambiguous description of the structural (fault) transfer zone” and the “insufficient ability of the supporting figures to illustrate the characteristics of the transfer zone,” we have carefully revised the manuscript and expanded the relevant sections following your suggestions.

Clarification of the spatial extent of the transfer zone

We have clearly indicated the distribution of the transfer zone within the study area in the revised Figure 6. Through detailed fault interpretation and paleogeomorphic reconstruction, the spatial extent and location of the transfer zone have now been explicitly delineated.

Integrated analysis from map-view and cross-sectional perspectives

According to your constructive advice, we further elaborated the relationships between the faults forming the transfer zone from both map-view and cross-sectional perspectives. In the map-view figure, we provide the precise location and geometric outline of the transfer zone. In the cross sections, we present the structural geometries and interactions among the relevant faults in detail, thereby illustrating more clearly the evolutionary process of the transfer zone and the spatial relationships among the associated faults.

Once again, we sincerely thank you for your careful and thoughtful review. We believe that these revisions significantly improve the clarity and completeness of our interpretation of the transfer zone. Should you have any further comments or suggestions, we would be more than happy to make additional revisions.

10. There are numerous issues with the language expression, grammar, and professional terminology in the manuscript, necessitating systematic polishing and revision. For instance, "source-sink" should be uniformly changed to "source-to-sink"; the term "Convergent" is relatively ambiguous, especially when used in the classification of source-to-sink systems; expressions like "down-truncated and up-lapped" are extremely vague; what does "near-same-direction" mean? There are also issues with the standardized expression of seismic reflection characteristics such as amplitude.

Thank you very much for your careful review of my manuscript and for the valuable comments you provided. In response to the issues you raised regarding language expression, grammar, and the use of technical terminology, I have conducted a thorough revision and polishing of the manuscript.

Regarding the term “source-sink,” which you suggested should be standardized as “source-to-sink,” I have carefully reviewed all related expressions and have unified them accordingly throughout the manuscript to ensure consistency. This revision aligns the terminology with established usage in the geological sciences.

Once again, I sincerely appreciate your detailed review and constructive feedback. I will continue to improve the quality of the manuscript in accordance with your suggestions, and I look forward to any further comments you may have.

11. The descriptions of the captions for most figures are overly simplistic, making it impossible to directly comprehend the intended meaning conveyed by the illustrations merely through the maps and captions. For instance, in Figures 12 to 14, despite presenting drilling, core, and seismic profiles for various sedimentary facies, the geological phenomena and interpretations presented in each figure are not elucidated. Furthermore, for the seismic or geological profiles involved, the origin of the profile location needs to be clearly indicated in the caption

Thank you very much for your thorough review and valuable comments on our manuscript. Regarding your suggestions that “the figure captions are overly simplified” and that “the geological phenomena and corresponding interpretations require clearer explanation,” we have carefully considered these issues and made the following revisions.

Refinement of figure captions:

In accordance with your suggestions, we have revised the captions of Figures 12 to 14 by adding more explicit descriptions of the geological features illustrated and their corresponding interpretations. We have aimed to make the captions more detailed so that readers can readily understand the content and purpose of each figure from the caption alone.

Clarification of section locations:

In response to your comment on explicitly marking “the origin of section locations,” we have clearly indicated the positions of all seismic and geological cross-sections in the captions of the relevant figures.

We believe that these revisions significantly enhance the clarity, readability, and professionalism of the figures. Thank you again for your constructive and insightful suggestions.
