# Supplementary material for: Source-to-sink coupling and spatiotemporal evolution during multiphase rifting: The Jurassic-Cretaceous Lishu fault depression, Southeastern Songliao Basin
Source: PLoS One. 2026 May 4;21(5):e0348267. doi: 10.1371/journal.pone.0348267 (PMC13138628; doi:10.1371/journal.pone.0348267)
Supplement: Attachment — Submitted filename: Response to Reviewers-2026.1.27.docx [file pone.0348267.s004.docx]

Response to Reviewers
We sincerely appreciate the reviewers for their thorough evaluation of our manuscript and for providing insightful comments that greatly assisted us in improving the quality of the paper. We have carefully addressed each comment, and our detailed responses are provided directly below each corresponding remark in red text.Once again, we would like to express our gratitude for your time and constructive review of our work.

（1）The current title is overly long, and beginning with "Study on" is redundant. It is recommended to revise it to a more concise and idiomatic expression. Some terms are also inaccurate.

We thank the reviewer for this constructive and helpful suggestion. The title has been revised to be more concise and idiomatic by removing the redundant phrase “Study on” and by refining several terms. In particular, the expression “multiphase rift basins” has been replaced with “multiphase rifting” to more accurately describe the geological process, and the terminology of the source-to-sink system has been standardized.

The revised title now reads:

“Source-to-Sink Coupling and Spatiotemporal Evolution during Multiphase Rifting: The Jurassic-Cretaceous Lishu Fault Depression, Southeastern Songliao Basin.”

（2） The abstract is information-dense yet overly condensed. Consider streamlining the content to more clearly highlight the key objectives, methods, findings, and implications. The transition between the results (①–⑥) is somewhat abrupt. A more coherent narrative would improve readability.

We thank the reviewer for this constructive and insightful comment. In response, the abstract has been substantially revised to improve clarity, readability, and narrative coherence. Specifically, the content has been streamlined to more clearly emphasize the research objectives, data and methods, key findings, and geological implications. The previously itemized and condensed results have been reorganized into a continuous narrative with smoother transitions, thereby enhancing the logical flow of the abstract. These revisions improve the accessibility of the abstract while retaining the essential scientific contributions of the study.

Location in manuscript: Revised Abstract, Lines 21–46.

（3） The literature review in the introduction is comprehensive but appears lengthy and unfocused. It could be more sharply focused on recent advances in source-to-sink modeling within rift basins.

We thank the reviewer for this valuable suggestion. In response, we have revised the introduction to focus more sharply on recent advances in source-to-sink (S2S) modeling in rift basins. The revised text now emphasizes:

1) Quantitative and process-based S2S modeling approaches, including forward stratigraphic modeling, high-resolution seismic analysis, well-log interpretation, and provenance data.

2) How multiphase fault growth, drainage reorganization, and accommodation variations control sediment routing, depositional system development, and basin-scale stratigraphy.

3) Recent case studies that demonstrate the application of these methods to lacustrine and continental rift basins, highlighting their relevance to predicting sand-body distribution and reservoir potential.

Location in manuscript: Revised Introduction, Lines 51–83.

（4） The methodological description is detailed but could benefit from a clearer step-by-step explanation, particularly regarding the integration of different data types (seismic, logging, core).

We thank the reviewer for this constructive comment. In response, we have reorganized and revised the Methods section to provide a clear, step-by-step description of our workflow, explicitly highlighting the integration of seismic, well-log, and core data.

Specifically:

Stepwise workflow: The methodology is now presented in five sequential steps: fault activity quantification, paleogeomorphology reconstruction, provenance analysis, transport channel identification, and statistical analysis of sedimentary fans.

Data integration: At each step, we explicitly indicate how seismic, well-log, and core/petrographic data are combined to ensure consistency and improve interpretive robustness.

Quantitative analysis: Fault activity rates, valley geometry, and sedimentary fan statistics are calculated based on integrated datasets, and potential uncertainties (e.g., depth conversion and seismic-attribute extraction errors) are addressed.

Visualization: Paleogeomorphology and source-to-sink systems are now reconstructed and rendered in 3D to clearly demonstrate spatial relationships.

These revisions improve clarity, reproducibility, and readability, ensuring that the integration of multiple data types and the overall methodological workflow are transparent.

Location in manuscript: Revised Methods section, Lines 126–190.

（5） The discussion section touches on topics like global rift basin comparisons and quantitative relationships, which feels somewhat fragmented and lacks a tight connection to the core issue raised in the introduction: "the coupling mechanisms and evolutionary models of the source-to-sink system in the Lishu Fault Depression." It is recommended to streamline or integrate sections 5.3 and 5.4, emphasizing discussions directly relevant to this case study.

We appreciate the reviewer’s insightful comment. In response, we have revised Sections 5.3 and 5.4 to streamline the discussion and improve its focus. Specifically:

Integration and simplification: Global rift basin comparisons and quantitative relationships are now summarized concisely and only included where they directly inform the understanding of source-to-sink system evolution in the Lishu Fault Depression.

Enhanced linkage to core research question: All discussion points are now explicitly connected to the coupling mechanisms and evolutionary model of the Lishu Fault Depression S2S system, emphasizing fault activity, sediment routing, and depositional system response under multiphase rifting.

Improved narrative flow: The discussion now progresses logically from regional observations to case-specific interpretations, ensuring that broader examples support, rather than distract from, the core findings.

These revisions enhance coherence, focus, and relevance to the primary objectives of the study.
Location in manuscript: Revised Discussion, Lines 601–693.

（6） The questions raised in the introduction, such as "how episodic rifting influences sediment stacking patterns" and "how to establish a source-to-sink coupling model," are addressed in the results and discussion, but the presentation could be more explicit. It is suggested to provide an itemized summary at the beginning of the discussion or in the conclusion section to directly respond to the scientific questions posed in the introduction.

We thank the reviewer for this valuable suggestion. In response, we have revised the Discussion and Conclusions to provide a concise and explicit summary of how our findings address the key questions raised in the Introduction. Specifically:

Episodic rifting and sediment accumulation: The Discussion now clearly summarizes how variations in fault activity, drainage reorganization, and accommodation space during multiphase rifting controlled sediment routing, depositional system development, and sedimentary body scaling.

Source-to-sink coupling model: We explicitly highlight the reconstructed quantitative relationships among sediment supply, transport pathways, and depositional systems, providing a clear framework for a coupled S2S model of the Lishu Fault Depression.

Enhanced narrative linkage: These summaries are now included at the beginning of the Discussion and in the Conclusions, ensuring that the manuscript explicitly closes the loop between the research questions posed in the Introduction and the study results.

These revisions improve clarity and directly demonstrate how the study addresses the scientific objectives.

Location in manuscript: Revised Discussion, Lines 602–617.

（7） The conclusion section largely repeats the results and lacks a concise synthesis of the "coupling mechanisms" and "evolutionary models."

We thank the reviewer for this valuable suggestion. In response, we have substantially revised the Conclusion section to emphasize the coupling mechanisms and evolutionary model of the source-to-sink (S2S) system in the Lishu Fault Depression.

Specifically:

Integration of results into mechanisms: Rather than listing results, the revised conclusion now clearly links fault activity, sediment supply, and depositional processes, highlighting how differential fault activity controls accommodation space, sediment routing, and sand-body distribution.

Basin evolutionary model: We summarize the basin evolution from early rifting through rifting–contraction to shallow-water expansive systems, emphasizing how these stages are controlled by the interaction of tectonics and sediment transport.

Quantitative S2S coupling: Statistical relationships between geomorphic parameters (valley width, depth, cross-sectional area) and depositional system scale are now explicitly presented, demonstrating measurable links between tectonics, transport, and deposition.

These revisions provide a concise synthesis of both the mechanistic controls and the evolutionary framework, directly addressing the scientific objectives raised in the Introduction.

Location in manuscript: Revised Conclusions, Lines 740–763.

（8） Some figures lack complete information or have unclear labeling.

We thank the reviewer for this helpful comment. In response, we have carefully revised all figures to improve clarity and completeness:

1) Figure labeling: All figures now include fully labeled axes, units, legends, and annotations to clearly indicate key features such as faults, sequences, and depositional systems.

2) Figure captions: Captions have been expanded to provide concise descriptions of the data, interpretations, and symbols used in each figure.

3) Supplementary details: Where necessary, additional insets or enlarged views have been added to highlight critical features that were previously difficult to discern.

4) Overall clarity: Color schemes, line styles, and symbol sizes have been adjusted to ensure that all figures are legible in print and on-screen.

These revisions ensure that all figures are self-contained, clearly labeled, and fully support the interpretations presented in the text.

（9） The manuscript contains noticeable grammatical errors, unnatural expressions, and inaccurate or inconsistent terminology. A thorough language polish by a native English speaker or a professional editing service is highly recommended.

Thank you very much for this constructive comment. We fully acknowledge that the previous version of the manuscript contained grammatical errors, awkward expressions, and some inconsistencies in terminology.

In response to this concern, the entire manuscript has been thoroughly revised and professionally edited by a native English speaker with experience in scientific writing. During this process, we corrected grammatical and syntactic errors, improved sentence fluency and readability, and carefully standardized technical terms to ensure accuracy and consistency throughout the manuscript.

We believe that these revisions have significantly improved the overall language quality and clarity of the manuscript, making it more suitable for publication. We sincerely appreciate the reviewer’s suggestion, which has helped us to substantially enhance the presentation of our work.
