# Supplementary material for: Source-to-sink coupling and spatiotemporal evolution during multiphase rifting: The Jurassic-Cretaceous Lishu fault depression, Southeastern Songliao Basin
Source: PLoS One. 2026 May 4;21(5):e0348267. doi: 10.1371/journal.pone.0348267 (PMC13138628; doi:10.1371/journal.pone.0348267)
Supplement: Attachment — Submitted filename: Response to Reviewers-2026.4.13.docx [file pone.0348267.s005.docx]

**Response to Reviewers**

We sincerely appreciate the reviewers for their thorough evaluation of our manuscript and for providing insightful comments that greatly assisted us in improving the quality of the paper. We have carefully addressed each comment, and our detailed responses are provided directly below each corresponding remark in red text.Once again, we would like to express our gratitude for your time and constructive review of our work.

1. Overall Assessment: This study successfully reveals the coupling relationship between source evolution, fault activity, and sedimentary sand bodies, providing an important basis for rift basin evolution and hydrocarbon exploration. The Abstract and Conclusion sections should be further refined and elevated to better summarize the distinct characteristics of each part of the study.

Thank you for your comment. In response to your review suggestions, we have revised the Abstract and Conclusions sections accordingly and supplemented the description of source-to-sink system evolution types during the rifting stages. Please see Lines 40–45 in the Abstract and Lines 774–782 in the Conclusions for details.

The added content in the Abstract is as follows:“During the initial rifting stage, steep-slope nearshore subaqueous fans and slope–fan delta systems developed. In the early phase of intense rifting, dominant patterns included steep slope–transfer zone–fan delta, axial slope–fan delta, and eastern gentle slope parallel fault-step zone systems. In the late phase, axial drainage systems prevailed, forming axial slope–braided river delta patterns. During the late rifting stage, gentle slope–braided river delta source-to-sink systems became dominant.”

The added content in the Conclusions is as follows: “During the initial rifting stage, sediment supply was partitioned, leading to the development of steep-slope nearshore subaqueous fans and a differentiated source-to-sink coupling pattern of steep slope–fan delta systems. In the early stage of intense rifting, sediment supply became convergent, and the dominant source-to-sink coupling patterns included steep slope–transfer zone–fan delta systems, axial slope–fan delta systems, and eastern gentle slope parallel fault-step systems. In the late stage, axial drainage systems became dominant, primarily forming axial slope–braided river delta coupling systems. During the late rifting stage, axial sediment supply was further strengthened, and the system was characterized by the development of gentle slope–braided river delta source-to-sink coupling patterns.”

2. Tectonic-Sedimentary Coupling: It is suggested to clarify how segmented fault activity drives the migration of depocenters, as well as the differentiation patterns of sedimentary facies between steep and gentle slope zones.

Thank you for your valuable comments. In response to your suggestions, we have added a discussion on how segmented fault activity evolution drives the migration of the depocenter, which has been incorporated into the section on fault activity analysis (see Lines 238–265). In addition, we have supplemented the description of the differential distribution patterns of sedimentary facies between the steep-slope and gentle-slope zones (see Lines 629–684).

3. Source Analysis: The source system analysis could be further streamlined. Focus on elucidating the evolutionary partitioning of source and ensure that the source evolution across different rifting stages is clearly articulated.

Thank you for your valuable comments. In the provenance system analysis section, we have summarized the sediment sources of different rifting phases, and the content has been further streamlined. We can clearly identify the changes in provenance for each rifting phase. Please refer to Lines 308–353 for details.

4. Discussion Section: The paper provides a detailed summary of the S2S evolution in the Lishu Fault Depression, emphasizing the coupling of "source system-transport pathway-depositional system." The Discussion should more explicitly define the coupling mechanisms between these three components.

Thank you for your valuable comments. In response to your suggestions, we have added a description of the coupling relationships among the provenance system, transport pathways, and depositional system in the Discussion section. Please see Lines 636–684 for details.

5. Figures: Please remove unused well locations from the figures (e.g., Fig 1, Fig 16, etc.).

Thank you for your valuable comments. In response to your suggestions, we have removed unnecessary well locations from Fig 1, Fig 16, and other relevant figures.

6. Scale Bars: A scale bar should be added to Figure 15.

Thank you for your comment. In response to your review, we have added a scale bar to Fig 15.

7. Schematic Model: In Figure 17 (the conceptual model), briefly indicate the specific types of S2S systems developed within various structural domains of the study area.

Thank you for your valuable comments. In response to your suggestions, we have revised Fig 17 to include the types of source–sink systems developed in different structural domains.

8. Quantitative Analysis: Regarding Table 2, previous studies have frequently discussed the relationship between paleo-valley parameters in the source area (width, depth, width-to-depth ratio, cross-sectional area) and the scale of corresponding fans in the depositional area. It is suggested that the discussion in this paper delve deeper into these relationships to strengthen the quantitative aspect of the S2S research.

We sincerely thank you for your comments. In response to your suggestions, we have added a discussion on the quantitative analysis of the source-to-sink system in the Discussion section. Please refer to lines 744–753 for the detailed revisions.

The detailed content is as follows: “Overall, the scale of depositional areas in multi-stage rift sedimentary systems is closely related to the characteristics of transport pathways, and is also partly controlled by the rift evolutionary stage. During the early rifting stage, deformation is dominated by lateral extension, and basin stretching is mainly controlled by fault activity, resulting in proximal deposition. Under conditions of stronger fault activity, the areal extent of fan systems tends to be relatively small. In contrast, during the intense rifting stage, sediment supply is sufficient and fault activity is further enhanced, leading to a significant increase in the axial extent of depositional systems. Therefore, the sedimentary area within source-to-sink systems is to a large extent governed by the tectonic phases of the rift basin, while fault activity intensity and stress orientation are the key factors controlling the architecture of the source-to-sink system.”
